# AMH regulates ovary size by counteracting the positive influence of clustered ovarian follicle growth

**DOI:** 10.1093/humrep/deag022

**Published:** 2026-02-26

**Authors:** Carmen Lim, Lynn Yiew, Nicholas J Anderson, Peter Smith, Laurel Quirke, Alan Carne, Urooza Sarma, Ryan Rose, Martha Nicholson, Christine L Jasoni, Ping Liu, Simone Petrich, Jenny Juengel, Michael W Pankhurst

**Affiliations:** Department of Anatomy, School of Biomedical Sciences, University of Otago, Dunedin, New Zealand; Department of Anatomy, School of Biomedical Sciences, University of Otago, Dunedin, New Zealand; Department of Anatomy, School of Biomedical Sciences, University of Otago, Dunedin, New Zealand; Reproductive and Developmental Biology, AgResearch Invermay, Puddle Alley, Mosgiel, New Zealand; Reproductive and Developmental Biology, AgResearch Invermay, Puddle Alley, Mosgiel, New Zealand; Department of Biochemistry, School of Biomedical Sciences, University of Otago, Dunedin, New Zealand; Department of Anatomy, School of Biomedical Sciences, University of Otago, Dunedin, New Zealand; School of Biomedicine, Robinson Research Institute, University of Adelaide, Adelaide, Australia; Genea Fertility South Australia, Adelaide, Australia; Awanui Labs, Dunedin, New Zealand; Department of Anatomy, School of Biomedical Sciences, University of Otago, Dunedin, New Zealand; Centre for Neuroendocrinology, University of Otago, Dunedin, New Zealand; Department of Anatomy, School of Biomedical Sciences, University of Otago, Dunedin, New Zealand; Brain Health Research Centre, University of Otago, Dunedin, New Zealand; Department of Women’s and Children’s Health, Dunedin School of Medicine, University of Otago, Dunedin, New Zealand; Reproductive and Developmental Biology, AgResearch Invermay, Puddle Alley, Mosgiel, New Zealand; Department of Anatomy, School of Biomedical Sciences, University of Otago, Dunedin, New Zealand

**Keywords:** anti-Müllerian hormone, ovarian follicle, prohormone convertase, microdialysis, ovary, spatial analysis

## Abstract

**STUDY QUESTION:**

Does anti-Müllerian hormone (AMH) influence preantral follicle development within a short range of AMH-secreting antral follicles?

**SUMMARY ANSWER:**

Immunization of sheep against AMH leads to increased follicle survival, specifically in regions close to small-medium antral follicles.

**WHAT IS KNOWN ALREADY:**

Serum AMH is known to inhibit the survival of immature ovarian follicles, but its biological role remains poorly understood. Mammalian ovaries contain many more developing ovarian follicles than are needed for ovulation, but how these systems operate remains unclear.

**STUDY DESIGN, SIZE, DURATION:**

Cross-sectional–control versus treatment studies examining the effects in the presence or absence of AMH.

**PARTICIPANTS/MATERIALS, SETTING, METHODS:**

Anti-AMH immunization experiments were conducted in sheep followed by histological 2D and 3D tissue analysis. Microdialysis was conducted on *ex vivo* human and sheep ovaries. Live-imaging of a fluorogenic enzyme-responsive reagent was conducted on *ex vivo* mouse ovaries. Organ/follicle culture was conducted on *Amh*^+/+^ and *Amh*^−/−^ ovary tissues to measure follicle activation and growth rates.

**MAIN RESULTS AND THE ROLE OF CHANCE:**

The likely site of action of AMH was shown to be ovarian stroma adjacent to large follicles as microdialysis determined that receptor-activating concentrations of AMH were only observed within a short range of the follicle. The AMH-activating enzymes were also shown to be primarily located in stroma using live-imaging of a fluorogenic enzyme-responsive reagent. Sheep were immunized against AMH protein to inhibit extracellular signalling and the ovaries were examined using 3-dimensional reconstructions of ovarian follicle positions. This showed that inhibition of AMH signalling caused an increase in preantral follicle survival, but almost entirely in proximity to large developing follicles.

**LARGE SCALE DATA:**

None.

**LIMITATIONS, REASONS FOR CAUTION:**

Limited studies were conducted on human tissue but the results concur with the sheep experiments. Sheep ovaries provide a useful large-animal model for comparative anatomy with humans but there will be interspecies differences.

**WIDER IMPLICATIONS OF THE FINDINGS:**

These results add to the evidence that small growing follicle survival is influenced by the proximity of large follicles. This has relevance for conditions where large follicles are lacking (e.g. primary ovary insufficiency) or where follicle growth is excessive (e.g. PCOS).

**STUDY FUNDING/COMPETING INTEREST(S):**

This research was funded by a Sir Charles Hercus Research Fellowship from the Health Research Council of New Zealand (grant number 18-027). The authors have no conflicts of interest to declare.

**TRIAL REGISTRATION NUMBER:**

N/A.

## Introduction

Anti-Müllerian hormone (AMH) is a TGFβ superfamily member first discovered as the foetal testicular hormone that removes the female reproductive tract (paramesonephric/Müllerian duct) from male embryos (MacLaughlin and Donahoe, 2004; Josso *et al.*, 2005). It was considered a male hormone for decades until AMH expression was observed in the adult ovary (Takahashi *et al.*, 1986) but its ovarian function remains unclear. The ovary is bestowed at birth, with a non-renewable supply of dormant (primordial) follicles that house the immature oocytes. Across the reproductive lifespan, primordial follicles are constantly activated to ensure a continuous supply of developing follicles is available for ovulation each ovarian cycle. As soon as a primordial follicle activates and begins development towards full maturity, AMH is expressed in the granulosa cells surrounding the oocyte (Baarends *et al.*, 1995). AMH expression increases throughout primary, preantral, and antral follicle development until the final days of folliculogenesis when the follicle prepares for ovulation and AMH synthesis tapers off (Jeppesen *et al.*, 2013).

Most ovarian hormones have clearly defined roles; estrogens are the primary sex hormones and orchestrate ovulation, progesterone facilitates pregnancy, inhibins regulate gonadotropin secretion, but a similar summary statement does not yet exist for ovarian AMH. AMH circulates in blood but the main site of AMH receptor (AMHR2) expression in adults is the granulosa cells (Takahashi *et al.*, 1986; Baarends *et al.*, 1995), suggesting that AMH might be primarily an autocrine signal, rather than a hormone. Paracrine AMH secreted from developing follicles is known to inhibit primordial follicle activation (Durlinger *et al.*, 1999), as the primordial follicles express AMH receptors but not AMH itself (Kano *et al.*, 2017). This function has been proposed to prolong the length of the female reproductive lifespan but *Amh*^−/−^ mice do not have reduced lifetime fertility and only have minor reductions in the timing of menopausal onset (Guo and Pankhurst, 2020). AMH also induces atresia (follicle degeneration) in early (preantral) follicle development (Pankhurst *et al.*, 2018; Zhou *et al.*, 2022) and reduces the production of estrogens in nearly-mature (antral) follicles (Xu *et al.*, 2016). Antral follicles require FSH to grow and survive (Kumar *et al.*, 1997) and some studies suggest that AMH inhibits this activity (Durlinger *et al.*, 2001; Hayes *et al.*, 2016), possibly to prevent follicle maturation from progressing too quickly (Jeppesen *et al.*, 2013). However, *Amh*^−/−^ mice ovulate functional oocytes from apparently normal mature follicles (Guo *et al.*, 2007; Visser *et al.*, 2007), suggesting that this is also not an essential role of AMH.

Perhaps the most confusing aspect of AMH signalling in the ovary is that it initially inhibits dormant primordial follicle activation (Durlinger *et al.*, 1999), then causes preantral follicle atresia (Pankhurst *et al.*, 2018; Zhou *et al.*, 2022), before switching back to trophic support as follicles transition from preantral to small antral and finally, becoming growth-inhibiting in large antral, and fully mature follicles (Xu *et al.*, 2016, 2018, 2020). We hypothesize that this allows small antral follicles to produce large quantities of AMH without ill-effect, and then secrete that AMH outside the follicle to cause atresia on nearby preantral follicles. Most AMH is secreted into follicular fluid as an inactive proprotein precursor (proAMH) (Pierre *et al.*, 2016) but at some stage before the AMH leaves the ovary and enters circulation, it is cleaved to the active form (Pankhurst and McLennan, 2013; Pankhurst *et al.*, 2016). This study aimed to determine the site of AMH activity in the ovary by examining locations of activity for the enzymes that cleave and activate proAMH, subtilisin/kexin type proprotein convertases (PCSKs) (Nachtigal and Ingraham, 1996). Finally, we aimed to examine 3D spatial interactions between the large follicles that produce the bulk of the AMH and the nearby preantral follicles that are susceptible to AMH-mediated atresia.

## Materials and methods

### Participants

Human ovary tissue was sourced from patients with *BRCA1*, *BRCA2*, or *RAD51* mutations undergoing prophylactic oophorectomy at Dunedin Hospital, Dunedin, New Zealand. Five patients were recruited between July 2019 and February 2021. Patients were eligible for recruitment if they were >18 years of age, premenopausal and had an indication for removal of one or both ovaries. Exclusion criteria were pregnancy, lactation, reproductive endocrine disorders, or endometriosis affecting the ovaries. Ovaries were transported immediately from the surgical theatre to the research laboratory for microdialysis measurements and sampling of ovarian follicular fluid. Microdialysis sampling was limited to 1 h to enable the ovaries to be transported to the pathology laboratory for fixation within 3 h of surgery. The sampling procedures had no effect on the quality of the subsequent histology and pathology assessments. No patients were found to have malignancies upon pathology examination. Ethical approval for this experiment was granted by the Health and Disabilities Ethics Committee, New Zealand Ministry of Health. All participants provided written informed consent.

### *In vitro* fertilization ovarian stimulation patient recruitment

Serum samples were obtained from 75 patients attending Fertility SA, St Andrews Hospital, Adelaide, Australia, a privately owned ART clinic to measure proAMH and total AMH levels during controlled ovarian stimulation. The mean (SD) age was 36.1 ± 4.8 years (range: 24–45). Standard gonadotrophin-releasing hormone antagonist stimulation cycles were used with doses of FSH individualized to each patient, based on ovarian reserve parameters, previous IVF response, or both. The FSH doses ranged from 100 to 400 IU. The first serum sample was obtained from patients when three follicles measuring wider than 17 mm were identified using transvaginal ultrasonography, and an hCG or agonist trigger injection was given within 24–48 h. A second blood sample was taken from each participant at the time of oocyte retrieval (36 h post-trigger injection). Immunoassays for proAMH and total AMH were performed as previously described (Pankhurst and McLennan, 2016). Of the 75 participants assay, only 15 had sufficiently high AMH levels for reliable quantification of the ratio of proAMH to total AMH and thus, inclusion in the final analysis (Total AMH >10 pmol/l or proAMH <0.9 pmol/l). The ratio was converted to an AMH prohomone index with the following formula: API=[proAMH]/[total AMH] × 100. Ethical approval for this experiment was granted by The University of Otago Human Ethics Committee (Health) and the St Andrew’s Hospital Human Research Ethics (STAND Project No. 93), Adelaide, Australia. All participants provided written informed consent.

### Animals

All studies comparing *Amh*^+/+^ and *Amh*^−/−^ mouse (Behringer *et al.*, 1994) ovary tissues utilized mice on a C57Bl6/J background. Adult mice were between 42 and 120 days of age and neonates were used at postnatal Day 2, where Day 0 was the day of birth. The animals were housed in a climate-controlled environment, with 12:12 h light/dark cycles and free access to water and standard rodent chow.

Sheep used in the AMH-immunization experiments were Romney breed aged between 2 and 5 years and all had given birth in at least one prior season. Sheep ovaries used in the microdialysis experiments were obtained from a local abattoir and were therefore, from mixed breeds.

All experiments on live animals or tissue from animals bred specifically for experimentation (i.e. not obtained from an abattoir) were either approved by the University of Otago Animal Ethics Committee or the AgResearch Animal Ethics Committee.

### Microdialysis

Sheep ovaries were obtained from the Silverfern Farms Finegand Abbatoir, Balclutha, New Zealand and were isolated immediately after the sheep had been slaughtered. The ovaries were transported back to the laboratory in Dunedin within 1 h, where they immediately underwent microdialysis. Human ovaries were obtained for the procedure, as described above. During microdialysis, ovaries were contained under an immersion buffer (0.9% w/v NaCl, 0.01 mol/l HEPES, 1% w/v BSA, pH 7.4).

AtmosLM™ microdialysis probes with a 1000 kDa molecular weight cut-off pore-size (Eicom, Cat No. PEP-12-04) were inserted into ovarian stroma adjacent to antral follicles estimated to be 2–5 mm in diameter. A 19-guage guide needle was first inserted with the microdialysis probe then lowered down through the needle. Perfusion buffer (0.9% w/v NaCl, 0.01 mol/l HEPES, 10% w/v BSA, pH 7.4) was pumped into the probe via a syringe pump and from probe to collection vessel via a peristaltic pump, with flow rate set to 2 µl/min. Microdialysate was collected over 1 h. At the end of the procedure, follicular fluid samples were taken using a 29-guage needle. Follicular fluid was centrifuged at 6700 ×*g* for 5 min to pellet out granulosa cells, then the supernatant was transferred to a clean tube and was frozen and stored at −80°C.

Microdialysis of recombinant human proAMH determined that the transfer efficiency into the microdialysate was 1.7%. AMH levels were assayed by ELISA (sheep: AMH (Ovine) ELISA, AnshLabs, Cat No. AL-155, human: Ultra-Sensitive AMH, AnshLabs, Cat No. AL-105) then the assayed values were adjusted by a factor of 58.8 to determine the stromal interstitial fluid AMH concentrations. The detection limit for AMH was 3.3 pmol/l in human follicular fluid and 10.5 pmol/l in sheep follicular fluid, after correction for extraction efficiency and sample dilution. Follicular fluid samples were diluted in assay buffer before AMH quantification by ELISA and correction for dilution factor.

### Active AMH immunization

Total cellular RNA was isolated from ovine ovarian tissue using TRIzol™ (Invitrogen) according to the manufacturer’s instructions. First-strand cDNA was synthesized from 5 µg of total RNA using SuperScript™ First Strand Synthesis System for RT-PCR (Invitrogen). The PCR primers (Forward: 5′ggatccgagcaccggagccgcggctgc and Reverse: 5′gaagcttccggcagccgcattcggtg) were designed to amplify the nucleotides encoding amino acids 467–575 of the ovine AMH (oAMH) protein (NP_001295528.1). The forward and reverse primers included BamHI and HindIII sites (underlined), respectively, required for subcloning into pQE31 expression vector. The PCR reaction employed the use of the GC-Rich PCR System (Roche). Following denaturation at 94 °C for 3 min, the cDNA was amplified using 35 cycles of denaturing for 1 min at 94 °C, 1 min of annealing at 60 °C, and extension at 72 °C for 2 min. A final extension was performed for 10 min at 72 °C. Amplification products were digested with BamHI and HindIII and subjected to electrophoresis through a 1% w/v low-melting point agarose gel. Product of the expected size was ligated into expression vector pQE31 (Qiagen) and the identity of the cloned insert confirmed by DNA sequencing. The recombinant plasmid was transformed into *E. coli* strain M15(pREP4) (Qiagen) followed by protein production induced by isopropylthio-b-galactoside and confirmed on western blots by labelling with anti-His antibody (Qiagen) and anti-AMH antibody (kindly provided by Nigel Groome). Induced cells were lysed by lysozyme treatment and sonification. The recombinant protein oAMH was solubilized in 8 mol/l urea and purified using Ni-NTA-agarose (Qiagen) utilizing the pQE31 C-terminal six-histidine His-tag vector sequence. Bound oAMH was eluted in the presence of 8 mol/l urea/250 mmol/l imidazole. Equal amounts of the *E. coli* produced oAMH and the carrier protein keyhole limpet hemocyanin (KLH) were conjugated in the presence of 1% w/v gluteraldehyde and 1 mol/l glycine. Conjugation was confirmed by SDS–PAGE.

Ewes were randomly assigned to groups either immunized with 0.4 mg of either KLH (control n = 6) or AMH-KLH (n = 5) in 1 ml of Freund’s complete adjuvant, given 4–5 months before the onset of the breeding season. The ewes then received five more injections every 28 days with 0.2 mg of KLH or KLH-AMH in 1 ml of sorbitan triolate, Tween 85, Marcol 52 mineral oil; 1:1:8 v/v/v. After the fifth injection, vasectomized rams with marking harnesses were run with the ewes to monitor estrous cycles with estrous cycle length calculated as the number of days between first and successive markings by the vasectomized ram. Ovulation rates were determined by laparoscopy over 3 cycles with final ovulation rate determined upon tissue collection after euthanasia. In addition, ovulation rate of all ewes was determined by laparoscopy 3–4 weeks prior to ovarian collection. Thus, the control ewes underwent laparoscopy two to three times and the treated groups most commonly once, although some were subjected to laparoscopy two to three times also. Ewes were euthanized using a captive bolt and exsanguination 30 days after the final injection. Ovaries were immersion fixed in Bouin’s fixative overnight at room temperature then were wax-embedded for haematoxylin and eosin histological staining of serial sections with 5 µm thickness. Sera was collected from all immunized ewes prior to immunization, 2 weeks after the booster immunization, and at time of ovary collection. Antibody titres were determined by qualitative ELISA. ELISA conditions were determined as follows: wells were coated with 100 µg/well of oAMH protein in 0.05 mol/l sodium bicarbonate buffer, pH 9.6; sera were diluted 1:5000; secondary antibody HRP-conjugated rabbit anti-sheep IgG (Invitrogen) was diluted 1:10 000. All AMH-immunized sheep sera contained anti-AMH antibodies 6 weeks after the first injection and at the time of euthanasia.

### Western blot

Recombinant PCSK3 (8 units) (New England Biolabs, Cat No. P8077L) was preincubated in 10 µl of 1.2 mmol/l CaCl_2_, 5 mmol MES (Merck, Cat No. 1.06126), pH 6.0 to activate the enzyme. Four picomole of recombinant human proAMH (PX’ Therapeutics) was added either in a buffer resembling the ion content and pH of extracellular fluid (1.8 mmol/l Ca^2+^, 140 mmol/l Na^+^, 4 mmol/l K^+^, 0.05% v/v Triton X-100 (brand), 100 mmol/l HEPES, pH 7.4) or one resembling conditions in the Golgi complex (Mellman *et al.*, 1986; Lissandron *et al.*, 2010) (0.4 mmol/l Ca^2+^, 12 mmol/l Na^+^, 107 mmol/l K^+^, 0.05% v/v Triton X-100 (Merck, Cat No. 648466), 100 mmol/l MES, pH 6.5). The final reaction volume was 40 µl and the incubation was conducted for 24 h at 37 °C. SDS–PAGE was run on 10% w/v Tris-glycine acrylamide gels with 4% w/v stacking gel at 100 volts for 1.5 h using the Xcell Surelock Mini-Cell system (Invitrogen). Proteins were transferred to a 0.4-µm nitrocellulose membrane (Whatman) at 30 volts for 1 h on ice. Blotting membranes were blocked with Odyssey blocking reagent (Licor) for 30 min then were probed with 0.1 µg/ml primary antibody to the N-terminal fragment of AMH (R&D systems, Cat No. AF2748, RRID: AB_2226475) then with 1 µg/ml IRDye 680RD donkey anti-goat IgG antibody (Licor, Cat No. 926-68074, RRID: AB_10956736). Immunofluorescence was imaged on an Odyssey infrared fluorescence scanner (Licor).

### Isoelectric focusing and 2D-PAGE

Aliquots (625 ng) of recombinant human AMH (produced in HEK293 cells by PX Therapeutics under contract to University of Otago, ∼20% proAMH, 80% AMH_N, C_) were combined with 130 µl of an isoelectric focusing (IEF) rehydration buffer [containing 7 mol/l urea, 2 mol/l thiourea, 2% w/v CHAPS, 50 mM DTT, 5 mmol/l TCEP (triscarboxyethylphosphine), to which 25 µl of IEF buffer concentrate (IPG buffer, GE Healthcare, Cytiva) was added per 0.5 ml of rehydration buffer, just prior to use], and the solution used to rehydrate overnight Immobiline DryStrip pH 3–10, 7 cm, linear gradient IEF strips (GE Healthcare). The rehydrated IEF strips were then subjected to electrophoresis in an IEF flat-bed electrophoresis system (IPGphor, Pharmacia Biotech) in a ceramic-welled plate with the IEF strips submerged under mineral oil, and using a voltage programme from 200 to 8000 V over 10 h, with a V/h target of 30 000 V/h. After IEF, the strips were applied to an in-house prepared second dimension SDS–PAGE (12.5% w/v acrylamide resolving gel, no stacking gel) and held in place using molten 0.5% w/v agarose. After electrophoresis, the SDS–PAGE gels were stained overnight with SimplyBlue SafeStain (Invitrogen) and then destained in water. An image of the destained gel was captured using a Canon CanoScan LiDE 600F scanner.

A second identical 2D SDS–PAGE gel underwent electroblotting to transfer the protein components to nitrocellulose membrane in transfer buffer (25 mmol/l Tris, 192 mmol/l glycine, 10% v/v methanol) at 300 mA for 3 h. Immunolabelling of the 2D blot was performed as described above. Densitometry measurements were performed using FIJI software (NIH) (Schindelin *et al.*, 2012; Rueden *et al.*, 2017).

### Protease-activated fluorogenic reagent imaging

Mouse ovaries from C57Bl6/J mice were dissected immediately after euthanasia. The ovaries were cut into fragments and were incubated in phenol red-free DMEM/F12 medium (ThermoFisher, Cat No. 11039021) containing 6 µmol/l Rh110 furin substrate (Sensolyte^®^ Rh110 Furin Activity Assay kit, Anaspec, Cat No. AS-72256), 66 µmol/l Alexa Fluor 555 Phalloidin (ThermoFisher, Cat No. A34055), and 1.6 mmol/l Hoechst 33342 DNA label (ThermoFisher, Cat No. H3570) at 37 °C, 5% CO_2_ for 2 h with gentle agitation every 30 min. The reagent is non-fluorescent but protease activity liberates fluorescent rhodamine 110. Rhodamine 110 accumulation of the incubated ovary fragments was quantified by confocal microscopy imaging with 488 nm excitation, 555 nm emission. Hoechst 33342 was imaged under 405 nm excitation, 450 nm emission. Fluorescence intensity was quantified using FIJI software using the ‘plot profile’ function. Pixel intensity of rhodamine 110 fluorescence was determined along straight-line traces intersecting with the centre of small-medium antral follicles. The angle of the line was predetermined by a random number generator. The mean pixel intensity of each zone was combined (µ_1_ = antral space, µ_2_ = granulosa, µ_3_ = theca, µ_4_ = stroma), and the total pixel intensity within the trace was calculated; µ_T_ = (µ_1_ + µ_2_ + µ_3_ + µ_4_)/4. For each follicle (n = 9), the mean pixel intensity of each zone was divided by µ_T_ to calculate a relative fluorescence unit (RFU) value. Repeated-measures ANOVA with Tukey’s B *post hoc* test was used to investigate differences in fluorescence across the anatomical zones.

### Neonatal ovary cultures

Neonatal mice were euthanized by decapitation and the ovaries were dissected from the bursa and were transferred into 2 µl media droplet on a transwell insert (0.4 μm pore size, Millicell, Merck, Cat No. PIHP01250) floating on 0.5 ml of DMEM/F-12 media, 100 U/ml penicillin/streptomycin (Gibco, Cat No. 1520096), ITS-G (Gibco, Cat No. 41400045) in a 24-well plate at 37 °C, 5% CO_2_. Cultures were treated with varied concentrations of recombinant human AMH_C_ (R&D Systems, Cat No. 17-37-MS-010) and were fixed in Bouin’s fixative for haematoxylin and eosin histological staining and follicle counting. Regression analysis was used to assess significant changes in primordial follicle inhibition. The results were plotted alongside response to recombinant AMH_C_ in a BMP-responsive element luciferase reporter assay conducted in P19 reporter cells according to previously described methodology (Kawagishi *et al.*, 2017).

### Neonatal ovary-adult follicle co-cultures

Neonatal ovaries were co-cultured with adult follicles (100–150 µm diameter) dissected from the ovaries of adult *Amh*^+/+^ and *Amh*^−/−^ mice with 30-guage needles. Follicles were placed adjacent to neonatal ovaries from *Amh*^+/+^ mouse pups and were cultured for 72 h. The cultures were observed daily and all adult follicles were confirmed to have become fused with the neonatal ovary within 24 h. The tissues were fixed in 4% w/v paraformaldehyde and were blocked with 5% w/v normal donkey serum (Merck, Cat No. D9663, RRID: AB_2810235), 0.2% v/v Tween-20 in phosphate-buffered saline for 3 h. Primary antibodies were 1 μg/ml mouse anti-DDX4 (Abcam, Cat No. ab27591, RRID: AB_11139638), 0.05 μg/ml goat anti-AMH (MIS C-20, Santa Cruz Biotechnology, Cat No. SC-6886, RRID: AB_649207) applied for 5 days at 4 °C. Secondary antibodies were 1 μg/ml donkey anti-mouse IgG-DyLight 488 fluorophore (ThermoFisher Scientific, Cat No. SA5-10166, RRID: AB_2556746), and 1 μg/ml donkey anti-goat IgG-DyLight 594 (ThermoFisher Scientific, Cat No. SA5-10088, RRID: AB_2556668) and 0.3 μmol/l of 4′, 6-diamidino-2-phenyllindole (DAPI) was included in the solution for a 4 day incubation at 4 °C. Z-stacks were obtained by confocal microscopy. Note that the DAPI fluorescence channel has undergone a digital linear adjustment to increase the brightness to a visible level in the displayed images.

### Follicle culture

A previously published method for follicle culture was adapted for these experiments (Hardy *et al.*, 2017). One follicle per well was cultured in round-bottom 96-well plates containing 100 µl of MEMα media with 100 U/ml penicillin/streptomycin (Gibco, Cat No. 1520096), ITS-G (Gibco, Cat No. 41400045) and 355 mIU/ml FSH (Genway, Cat No. GWB-3EE59E). Follicles were isolated from the ovary of *Amh*^+/+^ and *Amh*^−/−^ mice with 30-gauge needles with theca and some stroma still attached and were cultured for 96 h at 37 °C, 5% CO_2_. Follicles were divided into two groups for separate analysis based on initial diameter; 100–135 and 135–170 µm. Follicles were imaged for diameter measurement daily and 50% of the media in the well was replaced with fresh media once each day. Follicles were excluded from the analysis if there was a decrease in follicle size, loss of spherical morphology, or extrusion of the oocyte from the granulosa cell layer.

### Sheep ovary 3D follicle mapping

Sheep ovaries were bisected before embedding for histological sectioning. The whole ovary cross-section of every 20th section (100 µm section interval) was scanned at 20× magnification (Aperio CS2 Digital Slide Scanning System, Leica) and stored as .svs files. Observers were blinded to the treatments for follicle counting. Follicles were sub-classified into 1-layer (no more than 1 layer of granulosa cells at any point around the follicle), 1–2 layers (a second layer of granulosa cells starting to form but not completely enveloping the follicle), or 2–3 layers (a complete second layer of granulosa cells present but the third layer remaining incomplete). The 1 layer and 1–2 layer follicles were excluded if the cytoplasm was not visible and 2–3 layer follicles were excluded if the nucleus was not visible in the cross-section. The *x*, *y* coordinates from the centre of each follicle cross-section were recorded using the notation function in the ImageScope software (v12.4.3, Leica Biosystems Pathology Imaging). Coordinates obtained within individual images were designated *x*_u_, *y*_u_ and were recorded in a database. These data were also used to generate follicle counts, with an Abercrombie correction performed to account for differences in follicle diameter (Abercrombie, 1946).

Due to memory constraints, the .svs files could not be compiled into a Z-stack. Each .svs file had its resolution reduced to 72 dpi and was converted to a .tif file. The re-sized .tif files were loaded into Z-stacks in FIJI using the trackEM automated registration function to align the images correctly in 3D space. The Cartesian coordinates of the mid-point and diameter of all follicles larger than 100 µm in diameter (>3 layers) were determined and recorded in the stack. *Z*-coordinates were determined by multiplying the section number by 100 µm. These coordinates were stored in a separate database where the coordinates were designated *x*_3_, *y*_3_, *z*_3_. Large follicles were classed into specific size classes including 100–200 µm, 200–500 µm, 500–1000 µm, 1000–2000 µm, 2000–3000 µm, and >3000 µm. Follicles were recorded as atretic if the granulosa cells in the mural layer showed extensive pyknosis consistent with apoptosis and/or cellular degeneration of the oocyte. Atresia was not recorded in follicles with ≤3 layers.

**Figure 1.**
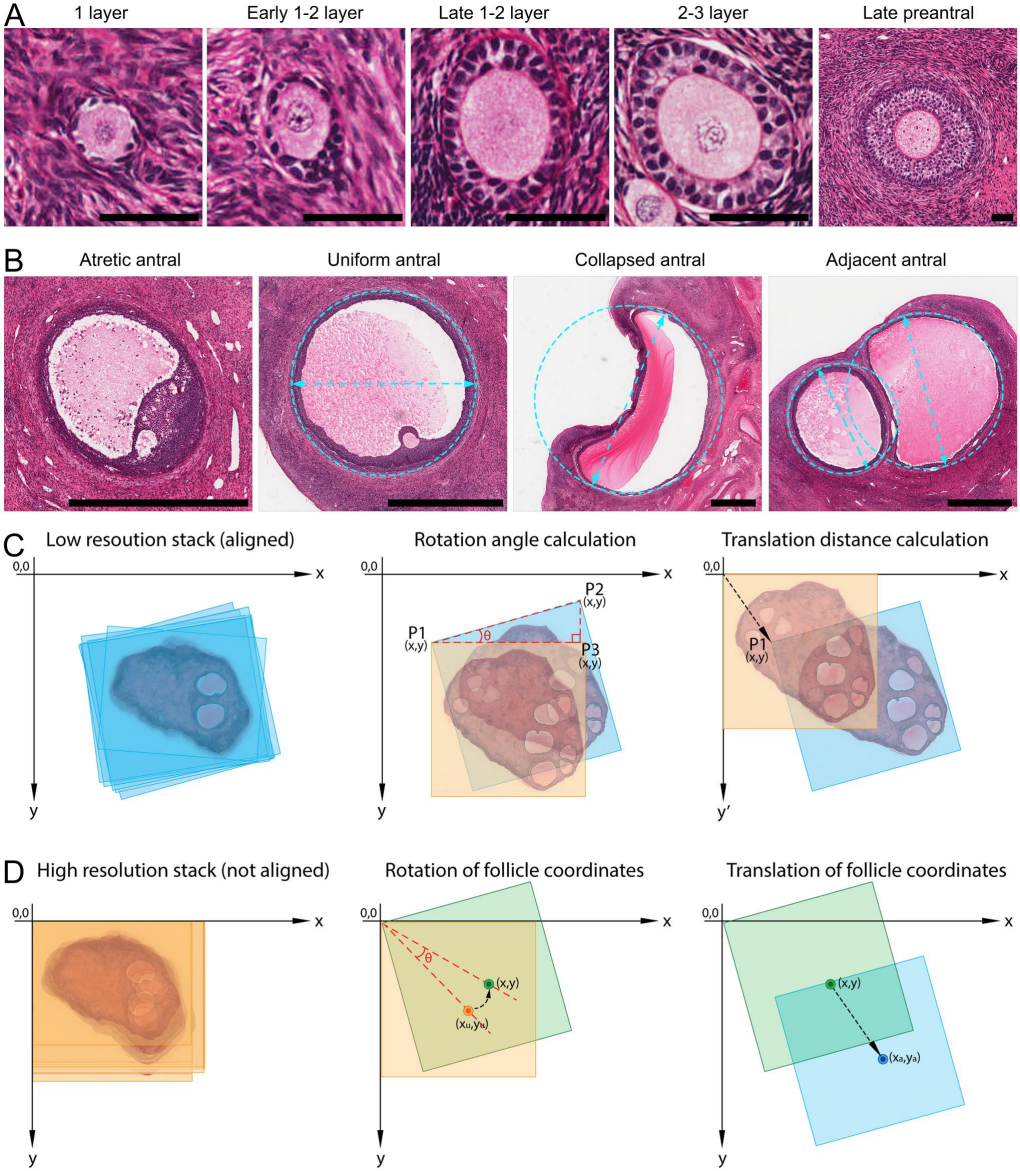
**Follicle classification and determination of 3D coordinates.** (**A**) Examples of follicles in different preantral stages of development based on the number of layers of granulosa cells. Late preantral follicles were recorded according to their diameter if they had more than three complete layers of granulosa cells (Scale bars = 50 µm). Follicle location was recorded as the *x*, *y* coordinate in the centre of the follicle. (**B**) Examples of antral follicles undergoing atresia or healthy follicles with uniform geometry, collapsed geometry caused by fixation or non-uniform geometry caused by follicles growing adjacent. Follicle diameters were determined at the widest point in the follicle and the midpoint was determined as the *x*, *y* coordinates at the centre of the line traversing the widest diameter of the follicle. The *z*-coordinate was determined from the section number multiplied by 5 µm. (Scale bars = 1000 µm). (**C**) Image resolution of all histological images was reduced to enable a full ovary image series to be loaded and aligned in a Z-stack in FIJI software for obtaining large follicle coordinates. The amount of image rotation used in the alignment was determined using trigonometry based on the new *x*, *y* coordinates of the top left (P1) and top right (P2) corners of the image. Translation was defined as the P1 coordinate as the top-left corner of an unaligned image is at the origin (0,0). (**D**) Determination of the *x*, *y* coordinates of small follicles requires high-resolution images that could not be loaded into a single Z-stack due to memory constraints. The coordinates of each small follicle were determined within individual images then the rotation and alignment steps were applied to all recorded *x*, *y* coordinates for small follicles. This step brings all small follicle coordinates in the database into alignment with the large follicle coordinates in 3D space.

The *x*_u_, *y*_u_ coordinates are accurate within each image but had not been aligned to the whole ovary *x*_a_, *y*_a_, *z*_a_ coordinates that were generated by the Z-stacks of the resized images. However, the re-sized images did not have sufficient resolution to identify preantral follicles. To resolve this problem, the *x*_u_, *y*_u_ coordinates generated from the .svs files were mathematically rotated to generate an intermediate *x*, *y* coordinate, which was then translated to align with the *x*_a_, *y*_a_ coordinates generated in the fully aligned low-resolution image Z-stack. The top left and top right corners of each image were designated P1 and P2, respectively (Fig. 1). P3 was generated from the *x*-value from P2 and the *y*-value from P1 to generate a right-angle triangle between the 3 points. The angle, θ, at P1 was calculated using trigonometry: cosθ=base length/hypotenuse length, where hypotenuse (distance between P1 and P2) and base (distance between P1 and P3) lengths were calculated using the formula for Euclidian distance:


$$d=\;\sqrt{{\left(x_{2}-x_{1}\right)}^{2}+{\left(y_{2}-y_{1}\right)}^{2}+{\left(z_{2}-z_{1}\right)}^{2}}$$

Within the .svs files, P1 represents the origin of the original image but within the Z-stack, P1 has been shifted by the alignment process. The *x*, *y* coordinates of P1 indicate how much the image needs to be translated, as this indicates how far the image has been moved from the origin. Therefore, the rotation and translation for each image in the aligned, re-sized .tif Z-stack were determined.

Each preantral follicle *x*_u_, *y*_u_ coordinate was rotated by angle θ to calculate the *x*_r_, *y*_r_ coordinates. The *x*, *y* coordinate of P1 was used to determine the translation distance to generate the *x*_a_, *y*_a_ coordinates (Fig. 1). The *z*_a_ coordinate was determined by the section number multiplied by section thickness (5 µm). The following formula was used for clockwise rotation and translation; *x*_a_ = (*x*_u_cosθ − *y*_u_sinθ) + *x*_P1_ and *y*_a_ = (–*y*_u_sinθ + *y*_u_cosθ) + *y*_P1_. An alternate formula was used for counterclockwise rotation and translation; *x*_a_ = (*x*_u_cosθ−*y*_u_sinθ) + *x*_P1_ and *y*_a_ = (*y*_u_sinθ + *y*_u_cosθ) + *y*_P1_.

Once all preantral follicle coordinates had been aligned with the coordinates recorded for large follicles, the data were rendered in 3D using Blender software v3.6 (The Blender Foundation). Data were imported with the Spreadsheet Importer Addon (Broggi, 2022). All follicles larger than 100 µm in diameter were generated with their true diameter. The 1 layer, 1–2 layer, and 2–3 layer follicles were assigned slightly larger diameters (60 µm, 120 µm and 160 µm, respectively) to improve their visibility in the rendered images and movies.

Distances between follicles were calculated using Euclidian geometry. The closest distance between the basal laminae of two follicles was calculated using the following formula:


$$d=\sqrt{{\left(x_{2}-x_{1}\right)}^{2}+{\left(y_{2}-y_{1}\right)}^{2}+{\left(z_{2}-z_{1}\right)}^{2}}\;-(r_{1}+r_{2})$$

where, *d* is distance, each set of *x*_a_, *y*_a_, *z*_a_ coordinates represents the midpoint of one follicles and *r* and is the radius of each follicle, from midpoint to basal lamina. The radius was set to zero for 1 layer, 1–2 layer, and 2–3 layer follicles, due to their small size and the difficulty in obtaining an accurate diameter measurement for individual follicles.

The distances between all follicles in the smaller classes (<200 µm) were determined relative to follicles in the larger classes (>200 µm). In total, 58 534 follicles from the control and AMH-immunized follicles were analysed. The analysis of small follicles (1 layer, 1–2 layer, 2–3 layer, or 100–200 µm follicles) within proximity of large follicles was limited to a zone reaching 2000 µm from the basal lamina of the large follicle. A maximum of 3000 data points were randomly selected from each follicle class in each ovary and pooled into control and AMH-immunized ovary groups for analysis. The data were analysed as a frequency histogram to examine which classes of small follicles could exist in proximity to larger follicles.

### Statistical analysis

Group differences between protease-activated fluorogenic signals across ovarian regions were compared with 2-tailed, 1-way ANOVA with Tukey B *post hoc* test. Group differences between primordial-primary follicle ratios in neonatal ovary-adult follicle co-cultures were compared with 2-tailed, 2-way ANOVA with Tukey B *post hoc* test. *Amh*^+/+^ and *Amh*^−/−^ follicle growth rates were compared with 2-tailed, 2-way repeated-measures ANOVA with LSD *post hoc* test and Levene’s test for homogeneity of variances. Differences in sheep ovary follicle counts were compared by 2-tailed Student’s *t*-test. Kolmogorov–Smirnov tests were used to examine whether distributions of distance between large follicles and 1-layer or 100–200 µm follicle were significantly different. The analysis was conducted within each treatment group for each of the large follicle size classes. Analyses were performed with SPSS v25 (IBM corporation) or Prism 5 (Graphpad software). Power calculations were performed with GPower (Faul *et al.*, 2007).

## Results

### AMH-signalling activity is strongest adjacent to the secreting follicle

**Figure 2.**
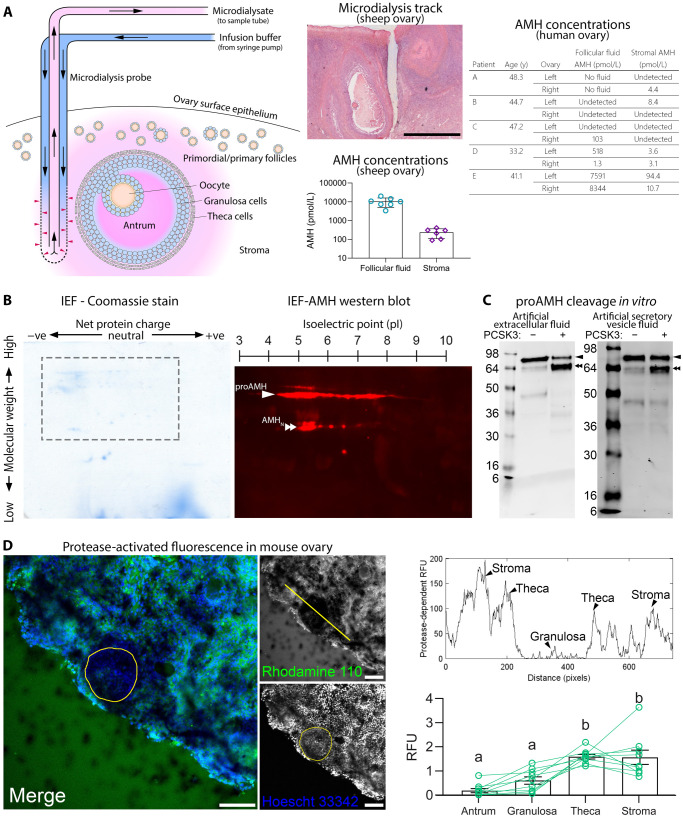
**Anti-Müllerian hormone (AMH) diffusion and proteolytic activation of proAMH in ovarian stroma.** (**A**) Microdialysis probes were inserted adjacent into ovary stroma where dialysis buffer was pumped through the porous probe allowing molecules up to 1000 kDa in molecular weight to enter the dialysate. The dialysate was collected for AMH assay. Needles were placed adjacent to an antral follicle as shown in the hematoxylin and eosin-stained sheep ovary section (Scale bar = 1 mm). AMH concentrations in follicular fluid or adjacent stroma were quantified in sheep and human ovaries. (**B**) 2D-PAGE separating recombinant AMH protein by size on the vertical dimension and isoelectric focusing (IEF) on the horizontal direction. Western blot was conducted on the dashed area using an antibody against the AMH N-terminal fragment (AMH_N_). The recombinant AMH preparation contains predominantly proAMH (arrow) and lesser quantities of AMH_N_. (**C**) Western blot of recombinant proAMH treated with (+) or without (−) recombinant PCSK3 in artificial extracellular fluid or artificial secretory vesicle fluid. ProAMH appears as a 72 kDa band (arrowhead) and the cleaved AMH_N_ fragment as a 64 kDa band (double arrowhead). (**D**) PCSK activity in mouse ovary fragments was visualized with a fluorogenic reagent that releases rhodamine 110 when exposed to protease activity (green: rhodamine 110 and blue: Hoescht 33342 DNA-stain, scale bar = 100 µm). A yellow ring denotes the basal lamina that separates the granulosa and theca layers. The upper graph shows the relative fluorescence intensity units (RFU) along the straight-line trace shown in the rhodamine 110 image and the lower graph shows mean (±SE) fluorescence intensity in the antrum, granulosa, theca, and stroma was compared with 1-way ANOVA (*F*_(3,32)_ = 15.8, *P* < 0.001) and Tukey’s B *post hoc* test (regions that share the same letter were not significantly different, n = 9).

Human antral fluid AMH concentrations have been reported to be as high as ∼9 nmol/l (Jeppesen *et al.*, 2013) and serum concentrations are usually below ∼50 pmol/l (Seifer *et al.*, 2011), but stromal AMH concentrations have not been quantified. Stroma concentrations were determined in sheep ovaries using microdialysis ranging from 90 to 410 pmol/l (Fig. 2A). This indicates that AMH becomes rapidly diluted as it diffuses out in ovarian stroma. The finding was also confirmed in human ovaries obtained after prophylactic oophorectomy (patient with cancer-causing *BRCA1/2* or *RAD51* mutations). Antral fluid AMH concentrations were very low in some patients, which is a feature of reproductive ageing (Robertson *et al.*, 2021). However, when follicular fluid levels were high, AMH could also be detected in the adjacent stroma, again with substantial dilution.

It remains unclear why most of the AMH in follicular fluid is uncleaved proAMH (Pierre *et al.*, 2016) but the site of extracellular cleavage could reveal information about the site of AMH action. We incubated recombinant proAMH with its cleavage enzyme, PCSK3 in an artificial extracellular fluid, leading to efficient cleavage (Fig. 2C). Cleavage was less efficient when the PCSK3 incubation was conducted in artificial Golgi apparatus/secretory vesicle fluid (Fig. 2C), suggesting that AMH is protected from intracellular cleavage and predisposed for extracellular cleavage. This experiment was repeated with replicates and densitometry revealed that the mean ± SE ratio of proAMH: AMH_N_ was 1.8 ± 0.2 in the artificial extracellular fluid and 3.8 ± 0.4 in the artificial Golgi fluid (*P* = 0.012, *t*-test). To determine the potential sites of proAMH cleavage, *ex vivo* mouse ovary fragments were incubated with a fluorogenic reagent that releases rhodamine 110 when exposed to PCSKs. The liberation of fluorescence was low in antral fluid and the granulosa layer but was high in stroma and theca (Fig. 2D, Supplementary Fig. S1). This observation is consistent with the conversion of proAMH to AMH_N, C_ in the theca and stroma and explains how AMH_N, C_ is present in circulation (theca is vascularized, granulosa is avascular).

The granulosa cells of ovarian follicles form a molecular sieve that allows small molecules (<100 kDa) to pass freely, but retains large molecules (>500 kDa) in the antrum to encourage osmotic fluid retention (Rodgers and Irving-Rodgers, 2010). Dimeric AMH has a molecular weight of 140 kDa and intermediate-size proteins (100∼150 kDa) can pass freely through the sieve if they carry a positive charge, but are restricted if they have a negative charge (Hess *et al.*, 1998). IEF to determine the net charge of AMH (Fig. 2B, Supplementary Fig. S2) showed variable protein charges, indicative of multiple post-translational modification variants. Densitometry indicated that 66% of the AMH was negatively charged, 18% was neutral, and 16% was positive. It is not clear why a large proportion of AMH carries a charge that would slow its egress from the follicle, but it may be a mechanism to prevent freshly generated AMH_N, C_ in the theca/stroma from diffusing back into the granulosa layer. Collectively, these experiments suggest that the target site of AMH activity is either the theca layer or the ovarian stroma immediately adjacent to the AMH-secreting follicle.

Prior evidence suggested that AMH activation is further influenced by LH (Bae *et al.*, 2008; Pankhurst and Chong, 2016). However, we examined the ratios of proAMH and total AMH in the serum of patients undergoing gonadotropin hormone stimulation for IVF and found no change after LH-receptor stimulation (Supplementary Fig. S3). The lack of change in proAMH to total AMH levels indicates that cyclic changes in gonadotropins are unlikely to affect rates of proAMH conversion to AMH_N, C_ across the ovarian cycle.

### Endogenous AMH has limited short-term effects on primordial follicles

**Figure 3.**
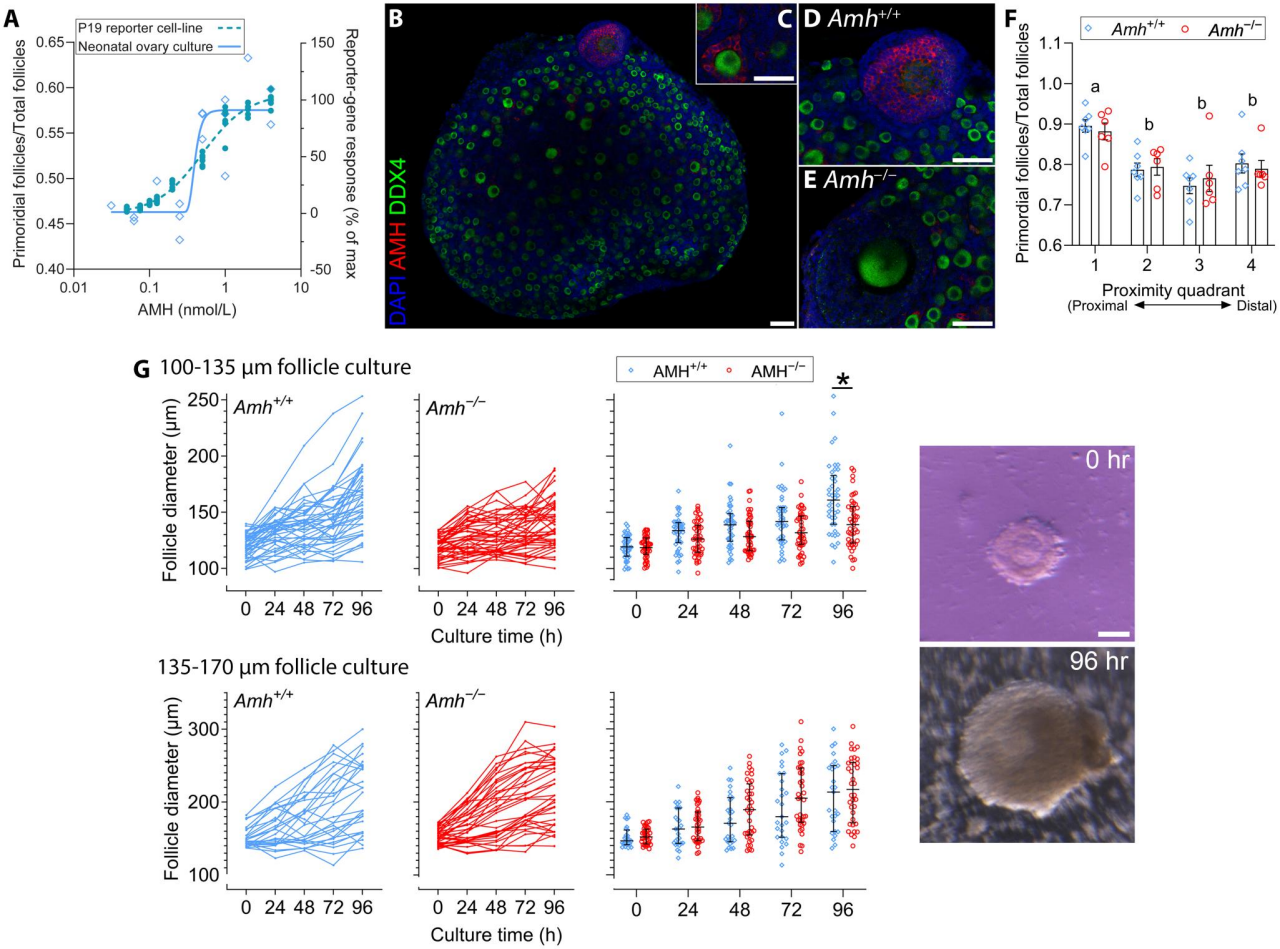
**The effects of anti-Müllerian hormone (AMH) on primordial and antral follicles.** (**A**) Dose–response curve of recombinant AMH on primordial follicle activation rates in neonatal mouse ovary cultures (blue open diamonds) and AMHR2-transfected P19 reporter cell line (green closed circles). Activation is expressed as the number of remaining primordial follicles as a fraction of all follicles (primordial + developing). (**B**) Confocal microscope images of antral follicles from adult *Amh*^+/+^ or *Amh*^−/−^ mice fused to *Amh*^+/+^ neonatal ovaries in co-culture with immunofluorescence for AMH, DDX4 (oocyte marker), and DAPI (DNA label). (**C**) Primary follicle expressing AMH in granulosa cells, (**D**) adult follicle from an *Amh*^+/+^ mouse, and (**E**) adult follicle from an *Amh*^−/−^ mouse, scale bars = 50 µm. (**F**) Distances between the antral follicle and all primordial or primary follicles in the antral follicle-neonatal ovary co-culture were divided into quadrants to investigate the primordial follicle activation rate at increasing distances from the antral follicle (2-way ANOVA with Tukey’s B *post hoc* test. Groups that share letters are not significantly different). (**G**) *Amh*^+/+^ and *Amh*^−/−^ small antral follicle growth in culture (* indicates *P* < 0.05, repeated measures ANOVA with Tukey’s B *post hoc* test) with representative images of follicles at 0 and 96 h *in vitro*.

In AMHR2-transfected transformed cell lines, the dynamic range of AMH activity occurs from 0.1∼4 nmol/l (Visser *et al.*, 2001; Kawagishi *et al.*, 2017). We treated neonatal ovary cultures with recombinant AMH_C_ and found inhibition of primordial follicle activation occurred over a similar range (Fig. 3A, *P *= 0.011, *r *= 0.615, regression), suggesting that ovarian tissues have the same dose–response curve. We then investigated whether endogenous AMH released from developing follicles was sufficient to induce the same effect. A single developing follicle derived from adult *Amh*^+/+^ or *Amh*^−/−^ mice was fused to an *Amh*^+/+^ neonatal ovary organ culture (Fig. 3B–E). We confirmed that dissected follicles still had the theca layer attached to ensure that PCSK3/5 expressing tissue was present to convert endogenous proAMH to AMH_N, C_. (Supplementary Fig. S4). Interestingly, the presence of an antral follicle was sufficient to significantly reduce primordial follicle activation rates in the adjacent area, when compared to more distant regions of the ovary (Fig. 3F, F_(1,3)_ = 10.1, *P *< 0.001, ANOVA). However, the effect occurred with antral follicles from either *Amh*^+/+^ or *Amh*^−/−^ mice. In this experiment, AMH did not have a strong influence beyond the other factors from antral follicles that influence primordial follicle activation (Fig. 3F, F_(1,3)_ = 0.19, *P *< 0.905, ANOVA), in contrast to the strong effects obtained with recombinant AMH (Fig. 3A).

### Endogenous AMH has limited short-term effects on small antral follicles

The effects of recombinant AMH on early antral follicle development have been contradictory, with both growth-promoting and growth-inhibitory effects observed, ever since the initial experiments (McGee and Hsueh, 2000; Durlinger *et al.*, 2001). The rapid drop in stromal AMH levels within a short distance of the follicle suggests that nearly all AMH found in a follicle is produced endogenously. We investigated the effects of endogenously produced AMH in late preantral or early antral follicles dissected from adult *Amh*^+/+^ or *Amh*^−/−^ mice (Fig. 3G). When late preantral follicles (100–135 µm diameter) were cultured for 96 h, *Amh*^+/+^ follicles grew slightly larger than *Amh*^−/−^ follicles (*F*_(1,4)_ = 11.7, *P *< 0.001, ANOVA). However, there was no difference in growth rates when early antral follicles (135–170 µm diameter) from *Amh*^+/+^ and *Amh*^−/−^ mice were cultured in the same manner (*F*_(1,4)_ = 1.4, *P *= 0.248, ANOVA). These relatively modest effects of AMH on primordial and late preantral/early antral follicles suggest that AMH might be part of a milieu of growth factors influencing follicle function but is not a strong regulator by itself.

### Antral follicle AMH induces atresia in immediately adjacent preantral follicles

**Figure 4.**
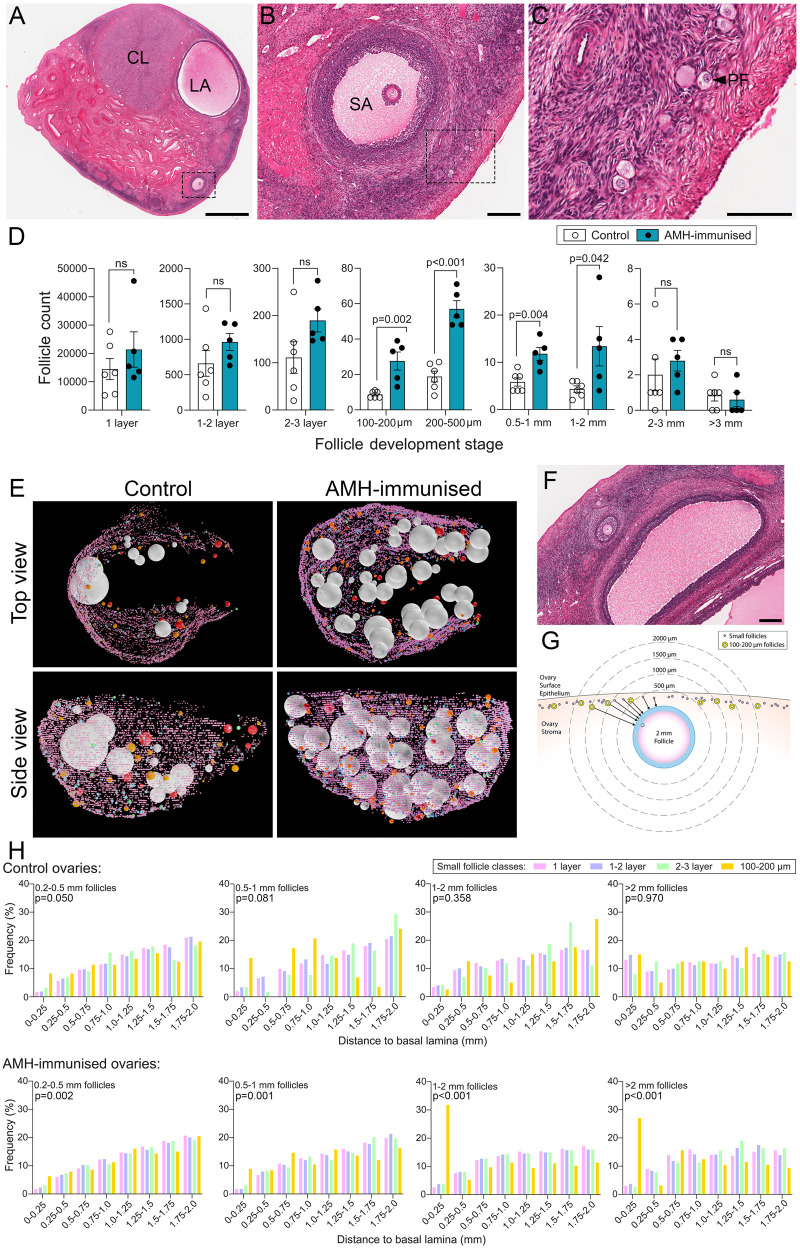
**Three-dimensional reconstruction of sheep ovary follicle locations.** All data shown were collected from half of a bisected ovary. (**A–C**) Haematoxylin and eosin-stained sheep ovary sections (CL; corpus luteum, LA; large antral follicle, SA; small antral follicle, PF; primordial follicle). The dashed line in panels (A) and (B) shows the region depicted in the subsequent panel. Scale bars: 2 mm, 200 µm, and 100 µm in A, B, and C, respectively. (**D**) Follicle counts in each ovary with small follicles classified by the number of granulosa cell layers (1 layer, 1–2 layers, or 2–3 layers) and large follicles classified by follicle diameter (*P*-values determined by Student’s *t*-test). (**E**) Follicle locations in 3D space in control and anti-Müllerian hormone (AMH)-immunized sheep ovaries. (**F**) Example of a 100–200 µm preantral follicle in proximity to an antral follicle in an AMH-immunized sheep ovary. Scale bar: 200 µm. (**G**) Schematic depicting follicle distance measurements between preantral and antral follicles. (**H**) Distributions of distances between large antral follicles and 1 layer, 1–2 layers, or 2–3 layers or 100–200 µm follicles within 2000 µm of the antral follicle basal lamina. Data are combined across all antral follicles from six control sheep ovaries or four AMH-immunized sheep ovaries. *P*-values were generated with Kolmogorov–Smirnov tests between the 1-layer and 100–200 µm follicles.

In our studies in mice, the most consistent effect of AMH that we have observed is the induction of atresia in early preantral follicles (Pankhurst *et al.*, 2018; Zhou *et al.*, 2022). We investigated this effect in a sheep model where animals were actively immunized against AMH protein or a vehicle–carrier protein, as a control. After 6 months of injections, the AMH-immunized sheep ovaries had no significant reductions in the smallest preantral follicles with 1, 2, or 3 layers of granulosa cells (Fig. 4D). However, counts of the largest (100–200 µm diameter) preantral follicles were significantly expanded in the AMH-immunized sheep compared to control ovaries. This difference persisted into the 200–500 µm follicle size range with the same ratio between the control and immunized sheep, indicating that no additional differences occurred in atresia rates as the follicles grew beyond 200 µm in diameter. This is very similar to the phenotype we observed in *Amh*^−/−^ mice (Zhou *et al.*, 2022). There were also no differences in mean ovulation rates between the AMH-immunized (1.68 ovulations per cycle), and control (1.68 ovulations per cycle) sheep.

Unlike mouse ovaries which are densely populated with follicles, the follicles in sheep ovaries were more dispersed around the ovary. It was therefore unclear how the removal of AMH signalling by immunization could affect preantral follicles across the whole ovary, when stroma concentrations suggest it can only act within a short distance of the follicle it is secreted from. To investigate this further, we mapped the 3D coordinates of 29 212 follicles from four AMH-immunized sheep ovaries and 29 322 follicles in six control ovaries (Fig. 4E, Supplementary Figs S5 and S6, Video 1). In control sheep, the follicles with 1 layer of granulosa cells (presumptive primordial follicle), 1–2 layers, or 2–3 layers were found distributed at all locations within the ovarian cortex. However, the 100–200 µm follicles were often found in proximity to larger follicles or in clusters with other similarly sized follicles suggesting proximity to other follicles highly increases late preantral follicle survival rates.

To determine if increased preantral follicle survival in AMH-immunized sheep was based on proximity to antral follicles, we calculated the distance between small follicles and large follicles and plotted the frequency distributions (Fig. 4H). Almost all of the increase in 100–200 µm preantral follicles could be found within 250 µm of the edge of a follicle larger than 1 mm in diameter (see example, Fig. 4F). The same relationship is seen when comparing the inverse; examining the distances between all 100–200 µm follicles and the nearest antral follicle larger than 1 mm in diameter (Supplementary Figs S7, S8, and S9). This indicates that AMH inhibits the excessive preantral follicle survival that occurs in close proximity to larger follicles.

## Discussion

**Figure 5.**
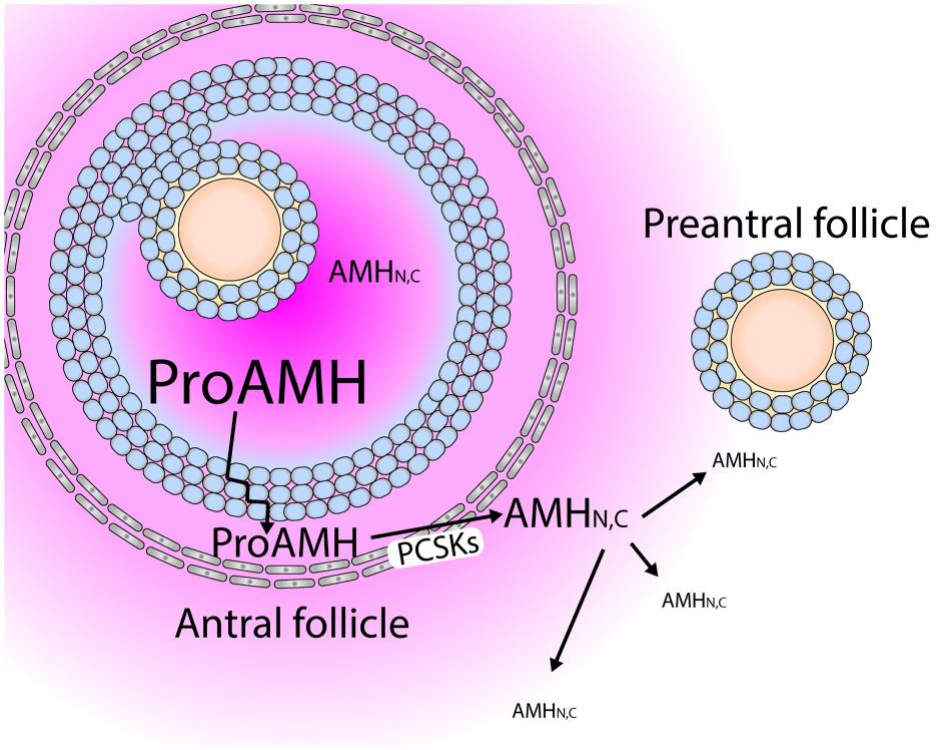
**Sites of pro-anti-Müllerian hormone (AMH) cleavage and conversion to active AMH_N, C_.** AMH is secreted into follicular fluid predominantly as the uncleaved, inactive proprotein, proAMH, with small amounts of the active form, AMH_N, C_. ProAMH diffuses through the granulosa layer slowly and will encounter increased prohormone convertase (PCSK) activity in the theca and stromal layers. The newly converted AMH_N, C_ becomes available to bind to receptors on cells/structures found in theca and stroma. Diffusion of AMH_N, C_ will preferentially occur into theca and stroma because diffusion back into the granulosa layer will be impeded by the net negative charge found on the protein.

In 2009, Da Silva-Buttkus *et al.* (2009), published an article predicting the existence of an unidentified local inhibitor of follicle growth in postnatal ovaries. Here, we use 3D spatial analysis to show that AMH is one such local inhibitor, and that these effects occur in adult ovaries, in large animals. Furthermore, this finding suggests that AMH action is required to balance the growth-promoting and survival-enhancing effects that antral follicles impart to nearby preantral follicles. We also showed that the enzymatic activity required for proAMH cleavage is highest in theca and stroma, which is consistent with inter-follicular signal transmission (see Fig. 5).

Microdialysis measurements in ovarian stroma indicated that AMH levels fell into the 0.1–1 nmol/l range within a short diffusion distance and our experiments in P19 cells and neonatal ovary cultures show that AMH concentrations below 0.1 nmol/l evoke minimal responses from the AMH receptor. This is consistent with actions of other TGFβ superfamily members and their activating proteases, which form diffusion gradients to determine how far their effects range from the site of production, particularly in developmental biology (Constam, 2014). It has been shown that proAMH can cause Müllerian duct regression, albeit at a slower rate than AMH_N, C_ (MacLaughlin *et al.*, 1992), which demonstrates that proAMH can be activated distant to the site of secretion and within target tissues. In ovaries, granulosa cells typically only express high levels of PCSK3 and PCSK5 in the preovulatory stages, where AMH expression in minimal (Bae *et al.*, 2008; Kelty and Curry, 2010). At less-advanced antral follicle stages, it is theca and surrounding stroma that produce the AMH prohormone convertases (Bae *et al.*, 2008; Kelty and Curry, 2010) and our new data show that this aligns with the location PCSK3 activity within ovarian tissue. This system may allow for a low level of AMH_N, C_ to mediate the functions of AMH within the follicle without overwhelming the AMH receptors. By contrast, the proAMH in follicular fluid may be specifically intended as a storage reservoir of AMH that is only activated once it leaves the follicle. The concentration of this ‘reservoir’ would need to be high to compensate for the rapid reduction in concentration that occurs with increasing diffusion distance.

Initially, it was not clear why large ovarian follicles would inhibit preantral follicle survival in such a short range. However, the AMH-immunization experiments showed that, when AMH signalling was inhibited, almost all of the increase in preantral follicle survival occurred immediately adjacent to large follicles. Developing follicles produce a range of growth factors that support follicular growth and survival (Field *et al.*, 2014) hence it is reasonable to assume that these factors would diffuse out of large follicles and influence the survival of surrounding small follicles. A recent study demonstrated this principle, showing ovary fragments containing large follicles can stimulate preantral follicle survival in ovaries rendered devoid of large follicles by chemotherapy (Batchvarov *et al.*, 2016). Antral follicles also promote angiogenesis in their thecal layer (Fraser, 2006), bringing an influx of nutrients and enhanced gas exchange, which may further improve growth conditions for nearby preantral follicles. In the absence of AMH to counterbalance this ‘proximity effect’, the preantral follicle population became substantially overgrown.

The ovaries of the average 20∼25-year-old woman will activate ∼450 primordial follicles per month (Wallace and Kelsey, 2010). If all follicles were allowed to reach 4 mm diameter (medium antral stage), their combined volume would be 15.1 cm^3^, which is larger than the average volume of both ovaries combined (∼9 cm^3^) (Hauth *et al.*, 2006). It takes an estimated 3 weeks for a follicle to grow from early-antral (2 mm), to the fully mature preovulatory size (20 mm) (Gougeon, 1996) and numerous follicles will attempt this each cycle. Therefore, the ovarian stroma needs to repeatedly accommodate large structural changes and presumably, the capacity of the ovary for cellular restructuring has an upper limit. This is evidenced by the accumulation of connective tissue in the ovary with ageing (Briley *et al.*, 2016) and one theory posits that elevated rates of mitosis in ovarian somatic cells may contribute to oncogenesis (Fathalla, 2013). Our recent research indicated that the majority of follicles are removed at the preantral stage when they are still relatively small (Zhou *et al.*, 2022), which would require less energy and cellular remodelling than removing large numbers of follicles at the antral stages.

One suggested role of AMH, based on *Amh*^−/−^ mouse studies (Durlinger *et al.*, 1999), is to inhibit primordial follicle activation and delay menopausal onset. However, we and others (Campbell *et al.*, 2012) did not observe an increased rate of primordial follicle activation in AMH-immunized sheep. Due to the high follicle density in mouse ovaries, most primordial follicles are in close proximity to antral follicles. In sheep, antral follicles are widely distributed and thus many primordial follicles would often be outside the range of an AMH-secreting antral follicle. It may be that the effects of AMH on primordial follicle activation are only observed at the whole ovarian reserve level in small or poly-ovulatory species. Alternatively, it is possible that time is required to see a cumulative effect AMH immunization on the ovarian reserve and that the 6-month duration of our experiment may have been insufficient.

Neonatal ovary or follicle explant cultures have been the standard method of assessing the actions of recombinant AMH (Durlinger *et al.*, 2002; Nilsson *et al.*, 2007). We were able to replicate this effect with recombinant AMH_C_, but we observed no effect of AMH when an adult antral follicle was fused to the neonatal ovary. This further demonstrates that follicular AMH output is not sufficient for long-distance influence over primordial activation and instead suggests that actions of AMH on primordial follicles are likely restricted to short ranges surrounding larger follicles. Interestingly, the presence of a nearby antral follicle was able to inhibit primordial follicle independent of the actions of AMH, suggesting that follicles exert other, stronger influences on primordial follicles. It is not yet clear if these effects were due to another soluble extracellular signalling molecule or whether the fibrous theca exerts influence via mechanotransduction (Shah *et al.*, 2018).

The role of AMH within follicles is also a topic of debate. AMH is often stated to inhibit the actions of FSH in antral follicles but we did not find evidence of this when comparing *Amh*^+/+^ and *Amh*^−/−^ follicle growth, which is the first experiment of this kind to examine the effects of endogenous AMH concentrations. The FSH-inhibitory effects of AMH have always been controversial, as even the first two studies showed opposing effects (Durlinger *et al.*, 2001; McGee *et al.*, 2001). In the present study, and other recent studies (Xu *et al.*, 2018, 2020), AMH seems to promote follicular growth from the late preantral stage into the small antral stage, which is not consistent with an FSH-inhibitory effect. AMH receptors have recently been detected in theca cells (Cheon *et al.*, 2018), and AMH has been shown to inhibit theca cell androgen production (Li *et al.*, 2020). This local androgen production appears to be important for preantral follicle growth and survival (Vendola *et al.*, 1998; Yang and Fortune, 2006; Laird *et al.*, 2017), hence these effects represent interesting avenues for further study of particular relevance to the mechanism of AMH-mediated preantral follicle atresia.

## Conclusions

The present study suggests that the primary role of AMH in the ovary is to prevent excessive numbers of follicles from surviving beyond the preantral stage. An important implication of these findings is that the composition of the follicle population in an ovary in the current cycle can influence the composition and structure of future cycles. This phenomenon could have important implications for ovarian ageing, infertility, and pathological conditions.

## Supplementary Material

deag022_Supplementary_Figure_S1

deag022_Supplementary_Figure_S2

deag022_Supplementary_Figure_S3

deag022_Supplementary_Figure_S4

deag022_Supplementary_Figure_S5

deag022_Supplementary_Figure_S6

deag022_Supplementary_Figure_S7

deag022_Supplementary_Figure_S8

deag022_Supplementary_Figure_S9

## Data Availability

The datasets generated during and/or analysed during the current study are available from the corresponding author on reasonable request.
