# Supplementary material for: AMH regulates ovary size by counteracting the positive influence of clustered ovarian follicle growth
Source: Hum Reprod. 2026 Feb 26;41(5):795–808. doi: 10.1093/humrep/deag022 (PMC13270314; doi:10.1093/humrep/deag022)
Supplement: deag022_Supplementary_Figure_S1 [file deag022_Supplementary_Figure_S1.pdf]

OXFORD
human reproduction
*Human Reproduction*, 2026, 1–1
https://doi.org/10.1093/humrep/deag022
Supplementary Data

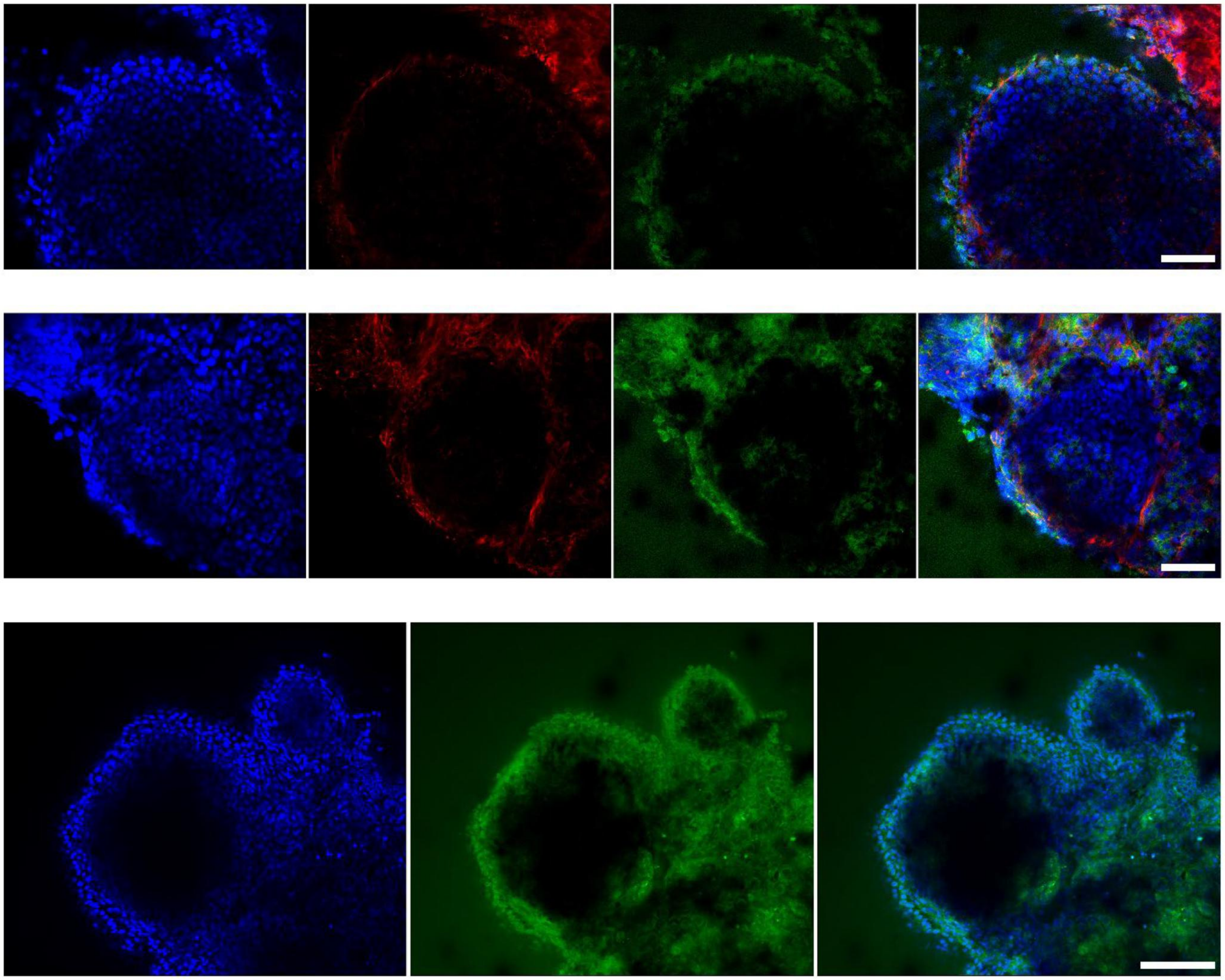

**Supplementary Figure S1. Fluorogenic protease activity labelling in *ex vivo* mouse ovarian tissue.** Blue: DAPI; Red: Phalloidin (F-actin labelling in theca and stromal layers); Green: protease-liberated rhodamine110. Colour-merged images have undergone linear intensity adjustments to balance the fluorescence intensities, single-channel images are unaltered. Top row scale bars = 50 μm. Bottom row scale bars = 100 μm.
