# Supplementary material for: AMH regulates ovary size by counteracting the positive influence of clustered ovarian follicle growth
Source: Hum Reprod. 2026 Feb 26;41(5):795–808. doi: 10.1093/humrep/deag022 (PMC13270314; doi:10.1093/humrep/deag022)
Supplement: deag022_Supplementary_Figure_S2 [file deag022_Supplementary_Figure_S2.pdf]

Human Reproduction, 2026, 1–1
https://doi.org/10.1093/humrep/deag022
Supplementary Data

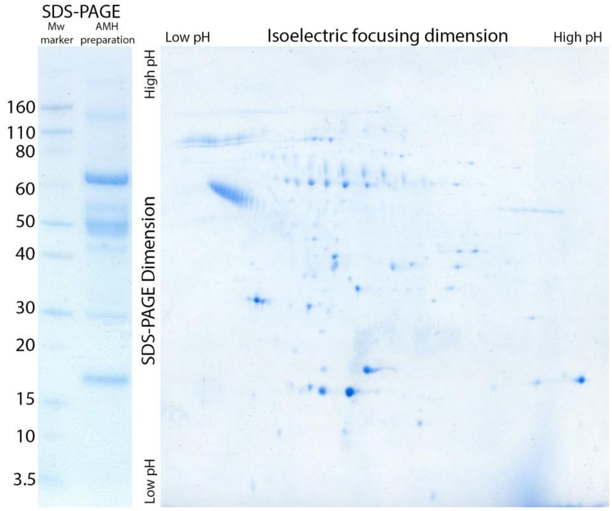

Supplementary Figure S2. Large 2-dimensional blot of the anti-Müllerian hormone (AMH) preparation (isoelectric focusing dimension horizontal, SDS–PAGE dimension vertical). The initial SDS–PAGE with molecular weight marker (kDa) is shown, left. The most prominent band at 72 kDa is the recombinant proAMH band.
