# Supplementary material for: AMH regulates ovary size by counteracting the positive influence of clustered ovarian follicle growth
Source: Hum Reprod. 2026 Feb 26;41(5):795–808. doi: 10.1093/humrep/deag022 (PMC13270314; doi:10.1093/humrep/deag022)
Supplement: deag022_Supplementary_Figure_S3 [file deag022_Supplementary_Figure_S3.pdf]

Human Reproduction, 2026, 1–1
https://doi.org/10.1093/humrep/deag022
Supplementary Data

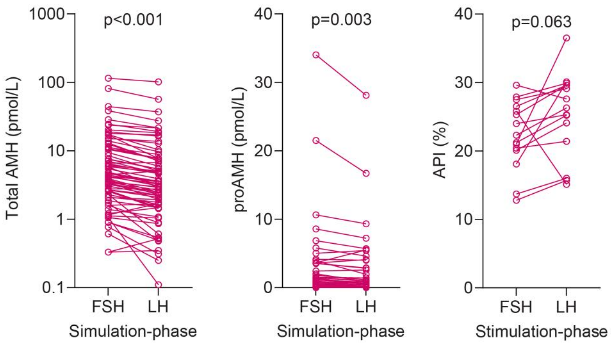

Supplementary Figure S3. Serum total anti-Müllerian hormone (AMH), proAMH, and the AMH prohormone index (API = [proAMH]/[total AMH] × 100) levels during controlled ovarian stimulation. Serum samples were obtained from patients undergoing controlled ovarian stimulation in both the FSH-stimulation phase and 24 h after human chorionic gonadotropin injection (which stimulates the LH receptors on ovarian follicles). A total of 77 patients were assayed for total AMH and proAMH but only 15 patients had sufficiently high total AMH or proAMH concentrations for accurate calculation of API values. Paired, 2-tailed *t*-tests demonstrate significant decreased in total AMH ($t_{(75)} = 5.499$, $P < 0.001$, effect size = −0.15, 95% CI: −0.46 to 0.17) and proAMH concentrations ($t_{(75)} = 3.058$, $P = 0.003$, effect size = −0.08, 95% CI: −0.39 to 0.24), but not the API ($t_{(14)} = 2.030$, $P = 0.06$, effect size = 0.54, 95% CI: −0.2 to 1.25), after LH-receptor stimulation.
