# Supplementary material for: AMH regulates ovary size by counteracting the positive influence of clustered ovarian follicle growth
Source: Hum Reprod. 2026 Feb 26;41(5):795–808. doi: 10.1093/humrep/deag022 (PMC13270314; doi:10.1093/humrep/deag022)
Supplement: deag022_Supplementary_Figure_S4 [file deag022_Supplementary_Figure_S4.pdf]

Human Reproduction, 2026, 1–1
https://doi.org/10.1093/humrep/deag022
Supplementary Data

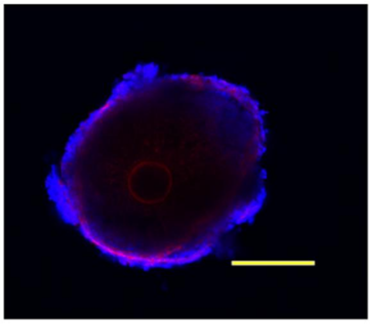

Supplementary Figure S4. Confocal optical section of a dissected adult ovarian follicle. Red phalloidin stain can be seen surrounding the oocyte and in the theca layer indicating that theca tissue was included in the dissected adult follicles. DNA is labelled blue with Hoescht 33342. A small amount of stromal tissue was attached to most follicles. These follicles were then fused to a neonatal ovary for the experiments shown in figure 3B–E.
Scale bar = 120 μm.
