# Supplementary figures and images for: AMH regulates ovary size by counteracting the positive influence of clustered ovarian follicle growth
Source: Hum Reprod. 2026 Feb 26;41(5):795–808. doi: 10.1093/humrep/deag022 (PMC13270314; doi:10.1093/humrep/deag022)

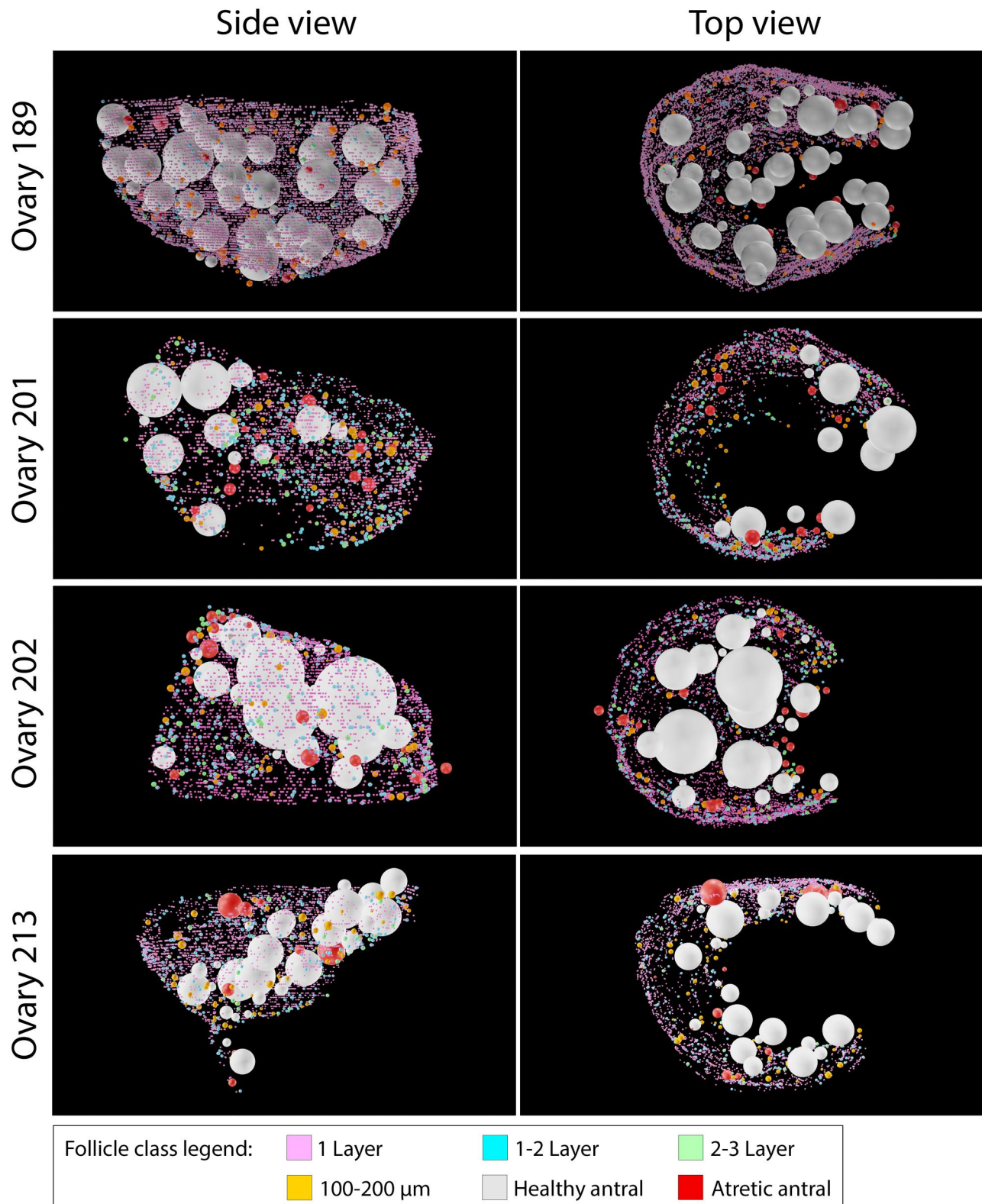

Supplementary Figure S6. 3D reconstructions of AMH-immunized sheep ovary follicle locations.

Supplement: deag022_Supplementary_Figure_S6 [file deag022_Supplementary_Figure_S6.pdf]
