# Supplementary material for: AMH regulates ovary size by counteracting the positive influence of clustered ovarian follicle growth
Source: Hum Reprod. 2026 Feb 26;41(5):795–808. doi: 10.1093/humrep/deag022 (PMC13270314; doi:10.1093/humrep/deag022)
Supplement: deag022_Supplementary_Figure_S7 [file deag022_Supplementary_Figure_S7.pdf]

Human Reproduction, 2026, 1–1
https://doi.org/10.1093/humrep/deag022
Supplementary Data

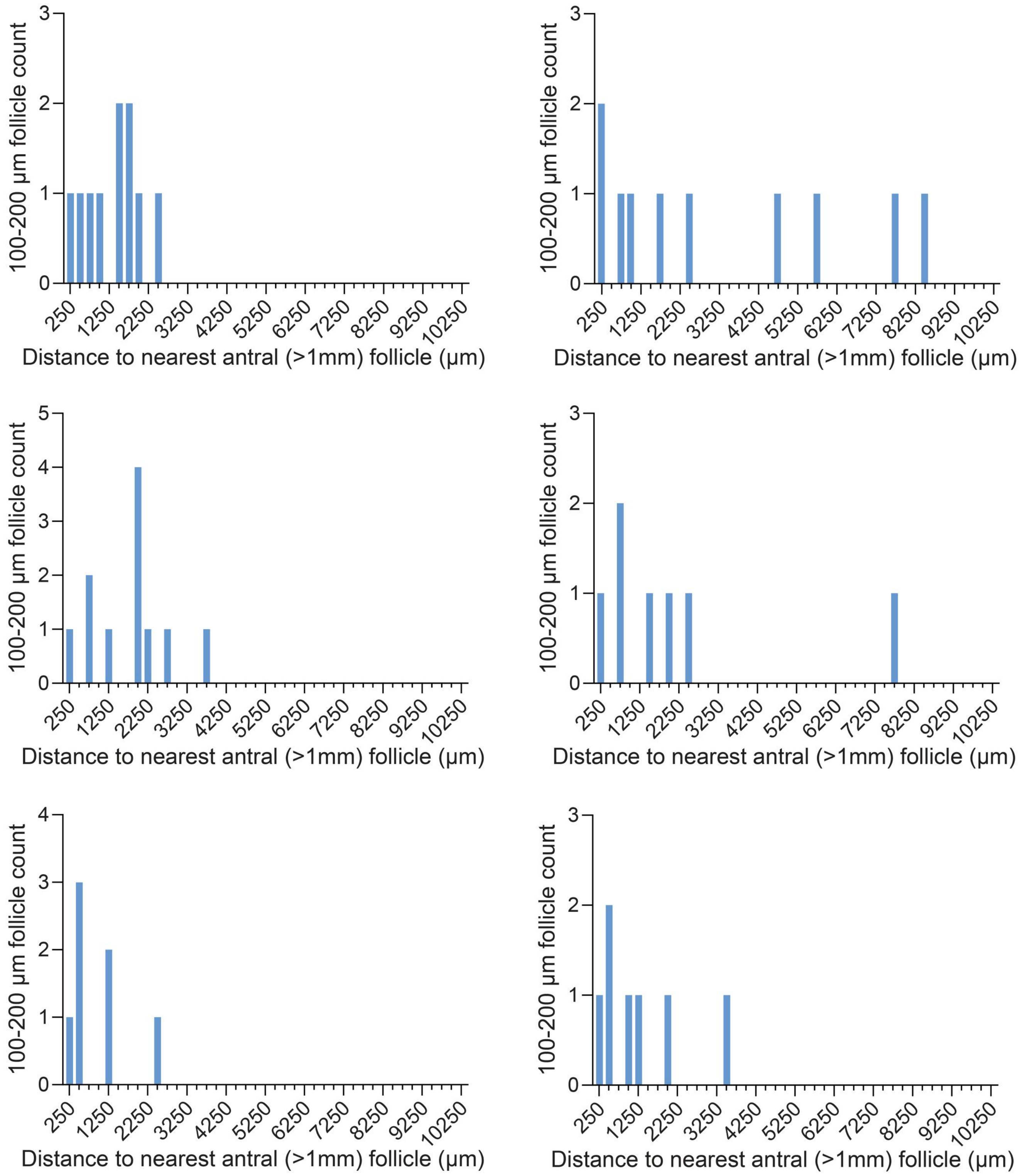

Supplementary Figure S7. Histograms of nearest large antral follicle neighbour to 100–200 μm diameter follicles in control sheep ovaries. For each 100–200 μm follicle, the distance to all follicles larger than 1 mm in diameter was calculated to determine the nearest distance to a large follicle. The histograms show how many 100–200 μm follicles fall within each 250 μm increment when considering the distance to the nearest large antral follicle. Each histogram represents data from one ovary. No 100–200 μm follicles were observed more than 10 mm distant from the nearest antral follicle >1 mm.
