# Supplementary material for: AMH regulates ovary size by counteracting the positive influence of clustered ovarian follicle growth
Source: Hum Reprod. 2026 Feb 26;41(5):795–808. doi: 10.1093/humrep/deag022 (PMC13270314; doi:10.1093/humrep/deag022)
Supplement: deag022_Supplementary_Figure_S9 [file deag022_Supplementary_Figure_S9.pdf]

OXFORD
human reproduction

Human Reproduction, 2026, 1–1
https://doi.org/10.1093/humrep/deag022
Supplementary Data

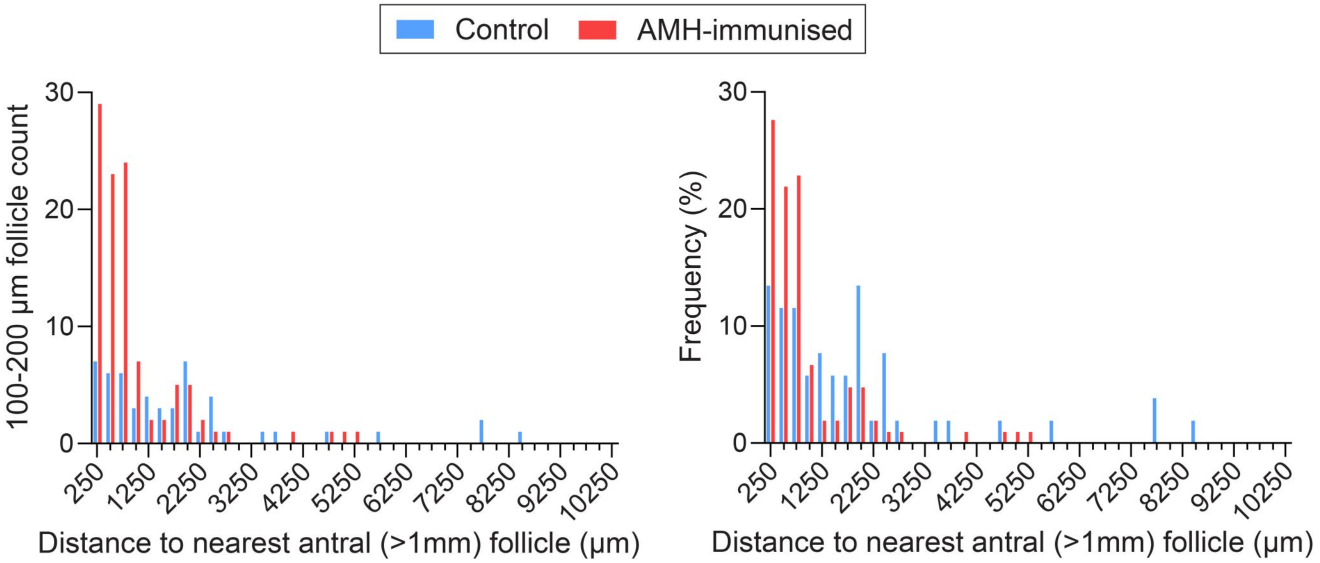

Supplementary Figure S9. Histograms of nearest large antral follicle neighbour to 100–200 µm diameter follicles combined across all control or anti-Müllerian hormone (AMH)-immunized sheep ovaries. For each 100–200 µm follicle, the distance to all follicles larger than 1 mm in diameter was calculated to determine the nearest distance to a large follicle. The histograms show how many 100–200 µm follicles fall within each 250 µm increment when considering the distance to the nearest large antral follicle. Each histogram represents data combined from all ovaries in the treatment-group showing either total follicle counts (left) or frequency relative to all data in the treatment-group (right). No 100–200 µm follicles were observed more than 10 mm distant from the nearest antral follicle >1 mm.

© The Author(s) 2026. Published by Oxford University Press on behalf of European Society of Human Reproduction and Embryology.
This is an Open Access article distributed under the terms of the Creative Commons Attribution License (https://creativecommons.org/licenses/by/4.0/), which permits unrestricted reuse, distribution, and reproduction in any medium, provided the original work is properly cited.
Human Reproduction, 2026, 00, 1–23
https://doi.org/10.1093/humrep/deag022
Original Article
